# Association between estimated time with low glomerular filtration rate and access to transplant among youth with advanced chronic kidney disease

**DOI:** 10.1007/s00467-026-07247-0

**Published:** 2026-04-06

**Authors:** Elaine Ku, Timothy Copeland, Charles E. McCulloch, Jonathan D. Savant, Bradley A. Warady, Susan L. Furth, Sarah Kizilbash, Daniel I. Feig, Roshan P. George, Joseph T. Flynn, Judith Jerry-Fluker, Matthew B. Matheson, Sandra Amaral

**Affiliations:** 1 Department of Epidemiology and Biostatistics, University of California San Francisco, 533 Parnassus Avenue MBU-E 414, San Francisco, CA 94158 USA; 2 Department of Medicine, University of California San Francisco, San Francisco, CA USA; 3 Division of Nephrology, Children’s Hospital of Philadelphia, Philadelphia, PA USA; 4 Division of Nephrology, Children’s Mercy Kansas City, Kansas City, MO USA; 5 Department of Pediatrics, University of Minnesota, Minneapolis, MN USA; 6 Department of Pediatrics, University of Alabama, Heersink School of Medicine, Birmingham, AL USA; 7 Emory University and Children’s Healthcare of Atlanta, Atlanta, GA USA; 8 Department of Pediatrics, University of Washington School of Medicine, Seattle, WA USA; 9 Division of Nephrology, Seattle Children’s Hospital, Seattle, WA USA; 10 Department of Epidemiology, Johns Hopkins Bloomberg School of Public Health, Baltimore, MD USA; 11 Department of Biostatistics and Epidemiology, Perelman School of Medicine, University of Pennsylvania, Philadelphia, PA USA

**Keywords:** CKD, Transplant, eGFR, Pediatrics

## Abstract

**Background:**

Slower chronic kidney disease (CKD) progression allows more time for transplant preparation. Whether differences in CKD progression by race/ethnicity associate with preemptive or living donor transplantation in youth has not been well studied.

**Methods:**

We examined the association between time spent with low eGFR (between 10–30 mL/min/1.73 m^2^) and odds of preemptive or living donor transplantation among youth with CKD. eGFR was estimated using the bedside Schwartz (if < 18 years) and CKD-EPI 2021 equations (if ≥ 18 years) and the CKiD U25 equation in sensitivity analysis. Time spent with low eGFR was compared by race/ethnicity.

**Results:**

Among 333 youth with CKD (median age 11 years [IQR 7,14]), median time spent with low eGFR was 28.8 months. 77% were preemptively waitlisted, and 45% received preemptive transplantation (56% of White and 24% of Black youth). Black (vs. White) youth had shorter time with low eGFR (–6.5 months; 95% CI –11.5, –1.4). Time with low eGFR did not differ across other groups. Findings were similar using the U25 equation. Every additional year spent with low eGFR was associated with higher odds of preemptive (OR 1.45; 95% CI 1.24–1.70) and living donor transplantation (OR 1.42; 95% CI 1.21–1.67), but not preemptive waitlisting (OR 0.96; 95% CI 0.83–1.11) in unadjusted analyses.

**Conclusions:**

Longer time spent with low eGFR is associated with greater odds of preemptive and living donor transplantation. Earlier transplant referral for all children, especially Black youth, may help improve access to preemptive and living donor transplantation.

**Graphical abstract:**

**Figure:**
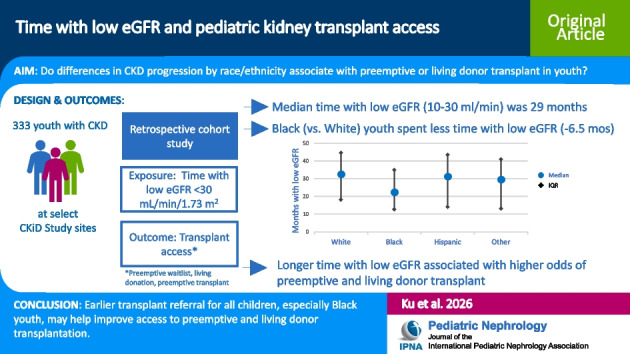
A higher resolution version of the Graphical abstract is available as Supplementary information

**Supplementary Information:**

The online version contains supplementary material available at 10.1007/s00467-026-07247-0.

## Background

Transplantation improves survival for those who reach kidney failure, yet non-Hispanic Black (NHB) and Hispanic youth are consistently less likely to receive preemptive transplantation and living donor kidney transplantation than non-Hispanic White (NHW) youth [1–6]. Intensive education about kidney replacement therapy (KRT) options does not usually begin until chronic kidney disease (CKD) stage 4 [7–9], and most guidelines recommend evaluating patients for transplant candidacy when the potential recipient’s estimated glomerular filtration rate (eGFR) has fallen below 30 mL/min/1.73 m^2^ [10]. Thus, the time spent with low eGFR (< 30 mL/min/1.73 m^2^) prior to the need for KRT is critical to the preparation of patients and their families for preferred kidney KRT modalities (such as transplantation) and identification of potential living donors.

In adults, NHB and Hispanic patients with CKD have been observed to experience more rapid progression to KRT compared with non-Hispanic White patients with CKD [11–19]. Similarly, in children, Black race has been noted to be a predictor of CKD progression, though the key drivers remain poorly understood and are hypothesized to be multifactorial, including gestational determinants, socioeconomic conditions and environmental exposures, in addition to biological factors like presence of APOL1 high-risk genotypes [20]. In adults, the more rapid CKD progression in Black individuals has been associated with shorter preemptive waitlist time accrual due to national policies which do not allow for waitlist registration to occur until eGFR is ≤ 20 mL/min [21, 22]. However, this policy does not apply to children < 18 years who can be waitlisted with no eGFR cutoff. Hence, whether time spent with low eGFR is a determinant of transplant access in children and young adults with pediatric-onset CKD (who could have been waitlisted prior to adulthood) is unclear, since waitlisting can occur at any time at the discretion of the pediatric provider and eGFR at listing is not required for pediatric kidney candidates. Furthermore, having an estimate of the potential differences in the timeframe over which children may reach kidney failure may help guide when to initiate transplant preparation. A recent editorial in *Pediatric Nephrology* highlighted the lack of national data on pediatric kidney waitlist and transplant practices in the USA as a major gap in knowledge to inform appropriate timing of transplant evaluation and waitlist registration in children with kidney failure [23]. To address this gap, we sought to quantify the magnitude of the difference in time spent with low eGFR by race/ethnicity in youth with CKD stage 4–5, and to confirm whether time spent with low eGFR is associated with the likelihood of preemptive and living donor transplantation or preemptive waitlisting.

## Methods

### Study population

The Chronic Kidney Disease in Children (CKiD) Study has enrolled > 1000 children between 1 and 16 years with an eGFR between 30 and 90 ml/min/1.73 m^2^ and has followed these participants prospectively since 2005 for kidney, cardiovascular, growth, and cognitive outcomes [24, 25]. The CKiD Study includes a nationally representative cohort and has active study sites across > 40 pediatric nephrology practices in the USA and Canada (Supplemental Table 1). Annual visits include data collection via surveys, physical exam, vital signs, and blood and urine specimens for centralized laboratory testing (including serum creatinine and urine protein/creatinine ratio). Ascertainment of the onset of kidney failure and death occurs routinely, and follow-up has continued even with the onset of kidney failure, regardless of the age of the patient. As a result, some young adults remained in the CKiD Study during follow-up.

In this CKiD ancillary study, children and young adults were eligible for inclusion in the analysis if they had at least 3 serum creatinine values and at least one eGFR (estimated by the bedside Schwartz equation) below 30 mL/min/1.73 m^2^ before they reached the need for KRT (dialysis or transplantation). Supplemental chart review to collect additional data elements at CKiD sites occurred in youth eligible for inclusion, including additional serum creatinine and height measurements and urine protein/creatinine or urine albumin/creatinine values approximately every 6 months starting 3 years prior to the onset of KRT. In addition, supplemental data regarding the date of waitlisting, initial dialysis treatment modality (hemodialysis or peritoneal dialysis), date of transplant, and donor source (living or deceased, if applicable) were also collected. Children’s Hospital of Philadelphia (CHOP) served as the single institutional review board (IRB) for the parent CKiD Study, providing centralized ethical oversight. In addition, local IRB review and approval were obtained where required by participating institutions. Informed consent/assent was obtained from participants and/or their legal guardians at time of CKiD enrollment, with re-consent as appropriate during subsequent study visits, in accordance with IRB guidelines and regulatory requirements.

Furthermore, to increase the sample size, we included eligible patients not enrolled in CKiD from 7 CKiD sites (University of California San Francisco, Seattle Children’s Hospital, Children’s Mercy Hospital, Children’s Hospital of Atlanta, Children’s of Alabama in Birmingham, University of Minnesota, Children’s Hospital of Philadelphia). These sites entered data retrospectively on youth < 25 years of age who were not CKiD participants but who met our inclusion criteria as noted above. Demographic, vital signs (specifically height), and laboratory data (including serum creatinine and urine protein/creatinine ratios) were collected to match the data collection instruments used in the CKiD Study and data were merged with CKiD data for pooled analysis. If a urine protein/creatinine ratio was not available, then dipstick albuminuria was accepted and converted to urine albumin/creatinine ratios using a validated equation [26]. If a urine albumin/creatinine ratio was available but a urine protein/creatinine ratio was not, values were converted using validated equations developed for each age group of interest [26, 27]. The most recent albuminuria prior to the start of time in models was used when possible, but if no value was available, the nearest value was used. Data were collected through the Research Electronic Data Capture (REDCap) system in de-identified fashion, and data collection was centralized at CHOP. This data collection was deemed not human subjects or exempt human subjects research at all participating sites by local IRBs, and the requirement for informed consent was waived.

Only children who had a decline in their eGFR (i.e., their final available eGFR was lower than their first available eGFR) were included. Additional inclusion and exclusion criteria applied to cohort selection are shown in Supplemental Fig. 1.

### Evaluating differences in the rate of loss of eGFR by race and ethnicity

The primary exposure was self-reported race and ethnicity (either collected by the CKiD study or from electronic health record systems) given that variable rates of progression of CKD have been described in children and adults.

The primary outcomes were the difference in time spent with eGFR 10–30 mL/min/1.73 m^2^, preemptive transplant, and living donor transplant by race/ethnicity. This time frame was selected to represent the time that would be available to prepare youth and their families for KRT. The endpoint of 10 mL/min/1.73 m^2^ was selected given that this is the median eGFR at start of dialysis among children in the USA [28]. Modeling time to a common eGFR (10 mL/min/1.73 m^2^) was applied as our primary analysis because of potential variations in timing of the start of dialysis and/or preemptive transplantation in clinical practice. However, in sensitivity analysis, we also modeled time spent with eGFR of 30 mL/min/1.73 m^2^ until the date of the actual start of KRT.

To determine the estimated time spent with advanced CKD (eGFR 10–30 mL/min/1.73 m^2^), we used a linear mixed modeling approach as previously described [21]. For this analysis, all eGFR values that were available were incorporated, including any available data collected from the CKiD study or the electronic health record systems. Mixed models were selected to accommodate the correlation between repeated values of eGFR within the same patient over time. This mixed model included linear and quadratic terms in time in the models as fixed effects (which were statistically significant) to capture non-linearities in the trajectory of eGFR. It also included individual-specific random intercepts and time trends as random effects to flexibly model individual trajectories.

We took several approaches to estimating GFR values included in the mixed linear regression models. First, we applied the bedside Schwartz equation when the participant’s age was < 18 years [29] and the CKD-EPI 2021 serum creatinine-based equation [30] when the participant’s age was 18 years or older, given that these equations were used in clinical practice in this manner for the vast majority of youth included in our analysis prior to the release of newer estimating equations. As an alternative approach, we applied the serum creatinine-based CKiD U25 equation [31, 32] to youth included for study. We did not have sufficient cystatin C data available from electronic health record systems to pursue estimates of GFR using cystatin C.

The primary models for eGFR trajectory used unadjusted mixed linear regression models since we were interested in repeated values of eGFR in the same individuals over time. We did not adjust for potential factors such as the cause of CKD or degree of albuminuria since these would be mediators of the differences in rates of loss of kidney function across differential racial groups, and because we were interested in quantifying absolute differences in estimated time spent with eGFR in youth with advanced CKD which is often the primary determinant of clinician decisions to initiate the transplant preparation or referral process.

To compare differences in estimated time with low eGFR between the Schwartz/CKD-EPI 2021 or CKiD U25 equations across racial groups, we employed a bootstrapping approach with 1,000 replications. This method allowed us to estimate confidence intervals around the difference in beta coefficients between the two separate regression models, which are not easily obtained through standard statistical approaches when comparing results across independent models.

### Determining whether time spent with low eGFR was associated with access to preemptive or living donor transplantation and preemptive waitlisting

Next, we used logistic models to examine the odds of preemptive kidney transplantation or living donor transplantation based on the estimated time spent with eGFR between 10–30 mL/min/1.73 m^2^. These outcomes were chosen because these are the preferred modalities of KRT for children, conferring the greatest graft and patient survival benefits [4]. We considered preemptive waitlisting a secondary outcome of interest. We included sequential adjusted models that were initially adjusted for age, sex, and cause of CKD (categorized as glomerular, congenital anomalies of the kidney or urinary tract, or other causes), and subsequently, models were additionally adjusted for urine albumin/creatinine ratios for these outcomes.

We tested for interactions between time spent with low eGFR and race/ethnicity (with p < 0.05 as the threshold for statistical significance) for each outcome separately, and finding the lack of an interaction, subgroup analyses were not pursued.

All analyses were conducted using STATA 17 (StatCorp TX, LLC).

## Results

We identified 333 youth who met our eligibility criteria (Supplemental Fig. 1). The median age of the cohort was 11 years (interquartile range (IQR) 7–14), 35% were girls, 22% were of Black race, 20% of Hispanic ethnicity, and 23% of the cohort had glomerular causes of their CKD (Table 1). The prevalence of glomerular disease as the cause of CKD was highest in those of Black race (32%), followed by those of Other race (31%) and Hispanic ethnicity (28%, *p* = 0.02, Table 1). A median of six serum creatinine measurements were available for incorporation into the mixed model trajectories. The median time between an eGFR of 10 and 30 mL/min/1.73 m^2^ was estimated to be 28.8 months (IQR 15.7, 42.5) when using a combination of the Schwartz equation with the CKD-EPI 2021 equations, and 31.8 months (IQR 17.3, 48.3) when using the CKiD U25 equation.

**Table 1:** Characteristics of patients by race/ethnicity

*n* (column %) or (25th & 75th percentile)	Total	White	Black	Hispanic	Other	***p*-**value
	333 (100.0%)	160 (48.0%)	72 (21.6%)	65 (19.5%)	36 (10.8%)	
Age at first measurement (years)	11 (7, 14)	10 (7, 13.5)	12 (9, 13)	12 (8, 15)	8.5 (4.5, 14)	0.23
Female	115 (35)	56 (35)	23 (32)	26 (40)	10 (28)	0.61
Cause of CKD						0.021
Glomerular	75 (23)	23 (14)	23 (32)	18 (28)	11 (31)	
CAKUT	89 (27)	51 (32)	18 (25)	15 (23)	5 (14)	
Other/unknown	169 (51)	86 (54)	31 (43)	32 (49)	20 (56)	
Number of eGFR measurements	6 (4, 9)	6 (4.5, 9.5)	6 (4, 7.5)	6 (5, 6)	6 (4, 11)	0.44
Lowest GFR (Schwartz & CKD-EPI 2021) in mL/min/1.73 m^2^	15.9 (11.7, 19.9)	16.1 (12.8, 20.1)	14.1 (10.2, 18.9)	15.2 (11.3, 20.0)	17.2 (12.7, 20.3)	0.065
Lowest GFR (CKiD U25) in mL/min/1.73 m^2^	16.0 (12.0, 20.1)	16.8 (12.8, 20.4)	14.2 (10.0, 18.2)	15.8 (11.0, 20.2)	16.2 (12.7, 20.7)	0.049
Estimated albumin-creatinine ratio (mg/g)	25.0 (1.4, 337.0)	43.3 (1.6, 396.9)	243.9 (1.0, 593.8)	12.0 (1.4, 316.2)	12.4 (1.6, 282.9)	0.49
Months between final eGFR and kidney failure	5.8 (4.4, 6.4)	5.6 (4.0, 6.3)	5.9 (4.9, 6.7)	5.9 (5.2, 6.2)	5.6 (3.3, 6.3)	0.18
Months from GFR 30 to 10 mL/min/1.73 m^2^ (Schwartz & CKD-EPI 2021)	28.8 (15.7, 42.5)	32.4 (18.2, 44.7)	22.3 (12.7, 35.0)	31.2 (14.1, 43.5)	29.5 (13.2, 41.0)	0.054
Months from GFR 30 to 10 mL/min/1.73 m^2^ (CKiD U25)	31.8 (17.3, 48.3)	35.9 (19.5, 50.4)	24.9 (14.0, 39.0)	31.8 (15.7, 50.8)	34.2 (14.3, 50.3)	0.030
Months between GFR 10 mL/min/1.73 m^2^ (Schwartz/CKD-EPI 2021) and actual KRT*	−3.0 (−9.8, 1.9)	−5.2 (−11.1, 0.5)	0.54 (−3.0, 5.9)	−2.0 (−10.8, 3.5)	−4.3 (−11.9, 0.9)	<0.001

*Negative values indicate estimated date of when eGFR is 10 ml/min/1.73 m^2^ occurs after KRT start; positive values indicate KRT occurs after estimated date of eGFR 10 mL/min/1.73 m^2^

When using the Schwartz/CKD-EPI 2021 equation, Black patients spent a mean of 6.5 fewer months (95% confidence interval (CI) –11.5, –1.4; *p* = 0.01) with low eGFR compared to White patients (reference group). Hispanic and Asian youth also spent fewer months with low eGFR than non-Hispanic White youth, but the magnitude of this difference was not statistically significant (Table 2). Findings were similar using the U25 equation (Table 2), though differences in time by race were larger, particularly for Black patients.

**Table 2:** Predicted months from eGFR 30 to 10 mL/min/1.73 m² using either a combination of Bedside Schwartz and CKD-EPI 2021 or CKiD U25

	Bedside Schwartz and CKD-EPI 2021			CKiD U25		
	*β* difference in months	95% CI	*p*-value	*β* difference in months	95% CI	*p*-value
Race						
White	Reference			Reference		
Black*	−6.5	[−11.5, −1.4]	0.012	−8.5	[−14.7, −2.3]	0.008
Hispanic	−0.8	[−6.0, 4.4]	0.76	−1.4	[−7.8, 5.0]	0.67
Other	−3.3	[−9.9, 3.2]	0.32	−2.8	[−10.9, 5.2]	0.49

*We determined the difference in the beta coefficients across the two separate models (using Schwartz/CKD-EPI eGFR versus U25 eGFR) by using a bootstrapping approach where the analysis was repeated 1,000 times to estimate confidence intervals around the difference in time. These 95% CI are not easily obtained otherwise since we are comparing differences in time across two separate models). Racial groups with an * had statistically significant differences (p < 0.05) across the Schwartz/CKD-EPI versus U25 models using this approach

When we examined the unadjusted time spent with low GFR modeled to the actual date of KRT onset (rather than modeling to an eGFR of 10 mL/min/1.73 m^2^), no statistically significant differences were noted in time spent with low GFR across all racial and ethnic groups (Table 3).

**Table 3:** Predicted months from eGFR 30 mL/min/1.73 m² to actual start of KRT using either a combination of Bedside Schwartz and CKD-EPI 2021 or CKiD U25

	Bedside Schwartz and CKD-EPI 2021			CKiD U25		
	*β* (months)	95% CI	*p*-value	*β* (months)	95% CI	*p*-value
Race						
White	Reference			Reference		
Black	−5.8	[−12.7, 1.2]	0.11	−8.4	[−17.5, 0.7]	0.07
Hispanic	−2.7	[−9.9, 4.5]	0.47	−5.7	[−15.1, 3.7]	0.23
Other	−1.6	[−10.7, 7.6]	0.74	−3.1	[−15.0, 8.9]	0.61

We determined the difference in the beta coefficients across the two separate models (using Schwartz/CKD-EPI eGFR versus U25 eGFR) by using a bootstrapping approach where the analysis was repeated 1,000 times to estimate confidence intervals around the difference in time. These 95% CI are not easily obtained otherwise since we are comparing differences in time across two separate models). No statistically significant differences across the Schwartz/CKD-EPI versus U25 models were identified using this approach

### Relation between time spent with advanced CKD and odds of preemptive or living donor transplantation

In terms of KRT access, 77% of youth were preemptively waitlisted, 10% were waitlisted after the initiation of dialysis, and 45% received a preemptive transplant (Table 4). Of those who received a preemptive transplant, 60% were from living donors. Most youth initiated KRT shortly after their last available eGFR measurement (median of 5.8 months [IQR 4.4, 6.4]; Table 1).

**Table 4:** Outcomes by race/ethnicity

*n* (column %)	Total	White	Black	Hispanic	Other	*p*-value
	333 (100.0%)	160 (48.0%)	72 (21.6%)	65 (19.5%)	36 (10.8%)	
Waitlisted						0.012
Not waitlisted	43 (13)	20 (12)	11 (15)	5 (8)	7 (19)	
Preemptively waitlisted	256 (77)	133 (83)	48 (67)	50 (77)	25 (69)	
Waitlisted	34 (10)	7 (4)	13 (18)	10 (15)	4 (11)	
Kidney replacement therapy						<0.001
In-center hemodialysis	122 (37)	46 (29)	37 (51)	22 (34)	17 (47)	
Home hemodialysis	4 (1)	1 (1)	1 (1)	2 (3)	0 (0)	
Peritoneal dialysis	58 (17)	21 (13)	16 (22)	17 (26)	4 (11)	
Preemptive transplant	149 (45)	92 (57)	18 (25)	24 (37)	15 (42)	
Donor type among transplants						<0.001
Living	89 (60)	65 (71)	4 (22)	13 (54)	7 (47)	
Deceased	53 (36)	24 (26)	10 (56)	11 (46)	8 (53)	
Does not remember	7 (5)	3 (3)	4 (22)	0 (0)	0 (0)	

Every one year longer estimated time spent with low eGFR was associated with a higher odds of preemptive transplantation when modeling eGFR decline using either the Schwartz/CKD-EPI 2021 equation (odds ratio (OR) 1.45; 95% CI 1.24–1.70) or U25 equations (OR 1.40; 95% CI 1.23–1.60, Table 5). These results were consistent in analysis using adjusted models (Table 5).

**Table 5:** Unadjusted and adjusted odds of preemptive transplant, living donor transplant, and preemptive waitlisting using either a combination of Bedside Schwartz and CKD-EPI 2021 or CKiD U25

	Preemptive transplant			Living donor transplant			Preemptive waitlisting		
	OR	95% CI	*p*-value	OR	95% CI	*p*-value	OR	95% CI	*p*-value
Unadjusted									
Years from GFR 30 to 10 mL/min/1.73 m^2^ (Schwartz & CKD-EPI 2021)	1.45	[1.24, 1.70]	<0.001	1.42	[1.21, 1.67]	<0.001	0.96	[0.83, 1.11]	0.59
Years from GFR 30 to 10 mL/min/1.73 m^2^ (CKiD U25)	1.40	[1.23, 1.60]	<0.001	1.35	[1.18, 1.54]	<0.001	0.97	[0.86, 1.09]	0.63
Adjusted*									
Years from GFR 30 to 10 mL/min/1.73 m^2^ (Schwartz & CKD-EPI 2021)	1.35	[1.13, 1.60]	0.001	1.34	[1.12, 1.61]	0.002	1.00	[0.84, 1.18]	0.99
Years from GFR 30 to 10 mL/min/1.73 m^2^ (CKiD U25)	1.33	[1.15, 1.53]	<0.001	1.28	[1.11, 1.49]	0.001	1.01	[0.88, 1.15]	0.94
Adjusted* additionally for log-transformed albuminuria									
Years from GFR 30 to 10 mL/min/1.73 m^2^ (Schwartz & CKD-EPI 2021)	1.31	[1.11, 1.56]	0.002	1.33	[1.11, 1.60]	0.002	0.99	[0.83, 1.17]	0.88
Years from GFR 30 to 10 mL/min/1.73 m^2^ (CKiD U25)	1.30	[1.12, 1.50]	<0.001	1.27	[1.09, 1.48]	0.002	0.99	[0.86, 1.14]	0.93

*Adjusted for age, sex, race, and cause of CKD

Similarly, longer time spent with low eGFR was associated with higher odds of preemptive living donor transplant with either the Schwartz/CKD-EPI 2021 equation (OR 1.42; 95% CI 1.21–1.67) or U25 equation (OR 1.35; 95% CI 1.18–1.54, Table 5).

However, the time spent with low eGFR was not associated with the unadjusted odds of preemptive waitlisting with either the Schwartz/CKD-EPI 2021 model (OR 0.96; 95% CI 0.83–1.11) or the U25 model (OR 0.97; 95% CI 0.86–1.09, Table 5).

No interactions between time spent with low eGFR and race/ethnicity were detected for the outcomes of preemptive waitlisting, preemptive transplantation, or living donor transplantation (all *p* > 0.05).

## Discussion

In this North American study of youth with CKD, we found that Black youth on average were predicted to spend 6–9 months less time with advanced CKD than non-Hispanic White youth when modeling time available to a common eGFR of 10 mL/min/1.73 m^2^. This finding persisted regardless of which equation was used to estimate GFR, but no differences were identified between Hispanic youth or youth of other racial backgrounds compared with White youth. Differences in time spent with low eGFR in Black youth were attenuated when modeling time to the actual date of KRT initiation. Longer time spent with low eGFR was associated with a higher odds of preemptive transplantation and living donor transplantation, but not with preemptive waitlisting. This finding was consistent regardless of the race/ethnicity of the patient.

Few studies in children have focused on the advanced CKD population given most national registries such as the United States Renal Data System only capture children at the time of KRT initiation [1, 3, 5, 23]. Additionally, the Scientific Registry of Transplant Recipients in the USA does not require eGFR at the time of listing for pediatric kidney transplant candidates [24]. Thus, there are no national data registries in the USA that include data on eGFR trajectories to inform clinical practice guidelines for timing referral, evaluation, and transplant surgery to avoid or minimize dialysis in children with advanced CKD. Interestingly, in our cohort, youth spent an average of more than two years with an eGFR between 30 and 10 mL/min/1.73 m^2^, though Black (compared to the White) youth spent 6–9 months less time with advanced CKD. We believe this is one of the first studies to quantify the magnitude of the differences in time spent with low eGFR in children with advanced CKD, and note that a one-year difference is clinically significant and could potentially explain the observed disparities in access to transplant by race/ethnicity shown in prior studies [33, 34]. Although there was a high prevalence of glomerular causes of CKD in Black youth (32%) and children with glomerular disease have been shown to have more rapid loss of kidney function, youth of other racial backgrounds (31%) and Hispanic youth (28%) had a similar high prevalence of glomerular disease as Black youth in this cohort [11], and yet only Black youth had substantially more rapid progression of CKD. Further studies are needed to identify the reasons for these findings.

Overall, these findings are consistent with those found in the Chronic Renal Insufficiency Cohort (CRIC) study in adults, which also showed that differences in the rate of progression were present by race/ethnicity [22]. The differential amount of time spent with low eGFR in the CRIC study was associated with a differential likelihood of waitlisting, which we did not identify in youth. However, this discrepancy likely stems from differences in national policies for waitlisting in adults and children [35], as adults cannot be waitlisted until their GFR is < 20 mL/min, whereas no such threshold exists for children. In addition, these findings suggest that access to waitlisting itself is less likely to be the source of disparities in access to kidney transplantation among youth, which we believe to be an important finding. We speculate that the longer time spent with low eGFR was more important for the attainment of preemptive transplantation than preemptive deceased donor waitlisting because preemptive transplant is highly driven by the availability of living donors whereas there are no eGFR criteria for deceased donor waitlisting children in the USA. It is likely that nephrologists recognize the multitude of benefits of reducing dialysis exposure and waitlist children promptly to accrue waiting time and benefit from pediatric priority allocation. Thus, children are listed inactively to accrue waiting time (without receiving organ offers), even when transplant may be sought through living donation or when transplant is deemed several years away. However, deceased organ access is still dependent on the national organ allocation system so it may be difficult to predict the time between transplant activation and receipt of a suitable, high-quality donor once a child truly needs transplantation. In contrast, preemptive transplantation (which most often occurs when living donors are available) is generally a complex process involving a high level of coordination of care and intentional planning. Preemptive transplantation with a living donor requires donor candidates to undergo an extensive workup and evaluation (which takes time) and recipients to be prepared for transplantation (which may also take time for vaccinations to be completed as well as recipient evaluation by subspecialists for comorbid conditions) as well as coordinated surgical planning and scheduling. Hence, the longer time spent with low eGFR would more strongly associate with preemptive transplantation than waitlisting.

The strong association between time spent with low eGFR and odds of living donor transplantation reinforces the importance of early transplant referral for youth, particularly those at risk for rapid loss of kidney function, to allow adequate time for identification and evaluation of living donors.

Although White youth were expected to spend 6–9 months longer with advanced CKD than Black youth, when we modeled to the actual date of kidney failure onset, there were no differences in time spent with low eGFR by race. A few possibilities could explain these findings. It is possible that Black youth start KRT at eGFR below 10 mL/min/1.73 m^2^, and hence the actual time to KRT may be longer than predicted, which is supported by the fact that the time between eGFR of 10 mL/min/1.73 m^2^ and actual date of onset of KRT was 0.54 months (Table 1). Alternatively, it is also possible that White youth undergo transplantation at higher eGFR, and hence their predicted modeled time to KRT is longer than the actual time (also supported by the fact that White patients started KRT a median of 5.2 months before their predicted time, Table 1). This would also be consistent with prior studies which have shown that dialysis and transplant occur earlier in White youth in the USA [36, 37].

There are several limitations in our study that should be highlighted. First, our findings may not generalize to all youth with CKD given that the centers contributing data are part of a national NIH longitudinal study, and care patterns and practices may differ from general practice. In addition, many of the centers included for study are large transplant centers. Nationally, 20–25% of children with incident kidney failure in the USA receive a preemptive transplant [38]. This percentage is substantially lower than what was observed in our cohort, suggesting that the children in our cohort may have had earlier access to transplant compared with children cared for in non-CKiD sites. It is thus particularly striking that even with two years of potential time with low GFR for children with kidney failure in this study, less than half (45%) of children were transplanted preemptively despite high rates of preemptive waitlisting (77%). Notably, preemptive transplant was largely driven by living donation.

Several other limitations should be noted. Many participating sites did not routinely ascertain proteinuria in youth with advanced CKD as part of routine clinical care, and some dipstick values and carry-forward imputation approaches were used to address this missingness, which may have led to misclassification of the severity of proteinuria. Additionally, we do not have data on barriers to preemptive transplantation or barriers to the identification of living donors. As in any observational study, residual confounding may be present and results may not generalize to all pediatric nephrology practices. Lastly, it is possible that we are unable to identify the presence of effect modification due to the more limited sample size of youth included for study, though we strongly believe that this is one of the largest cohorts of children with advanced CKD who have been well-characterized during longitudinal follow-up.

In conclusion, Black youth with CKD spend 6–9 months less time with low eGFR than other racial and ethnic groups, regardless of which equation was applied for estimating GFR; earlier preparation of Black children for transplantation may be warranted given the inequities observed in time spent with low eGFR, with particular attention to optimizing resources to support access to living donors. Time spent with low eGFR was strongly associated with access to preemptive and living donor transplantation, but not preemptive waitlisting, regardless of the individual’s race/ethnicity. These findings suggest that failure to preemptively waitlist youth is unlikely to be the mediator of the known disparities in access to kidney transplantation in youth. Earlier referral to transplant, with particular attention to allow time to identify living donor candidates, may be an important step to address the known disparities in access to timely preemptive transplant in youth with CKD.

## Supplementary Information

Below is the link to the electronic supplementary material.

Graphical Abstract (PPTX 81.5 KB)

ESM2 (DOCX 29.2 KB)

ESM3 Supplemental Fig. 1 Consort diagram (PDF 61.1 KB)

## Data Availability

The data were collected under data use agreements that do not allow for direct sharing of the data.
