# Supplementary material for: Association between estimated time with low glomerular filtration rate and access to transplant among youth with advanced chronic kidney disease
Source: Pediatr Nephrol. 2026 Apr 6;41(9):2979–88. doi: 10.1007/s00467-026-07247-0 (PMC13178790; doi:10.1007/s00467-026-07247-0)
Supplement: Supplementary file 1 — (PPTX 81.5 KB) [file 467_2026_7247_MOESM1_ESM.pptx]

## Slide 1
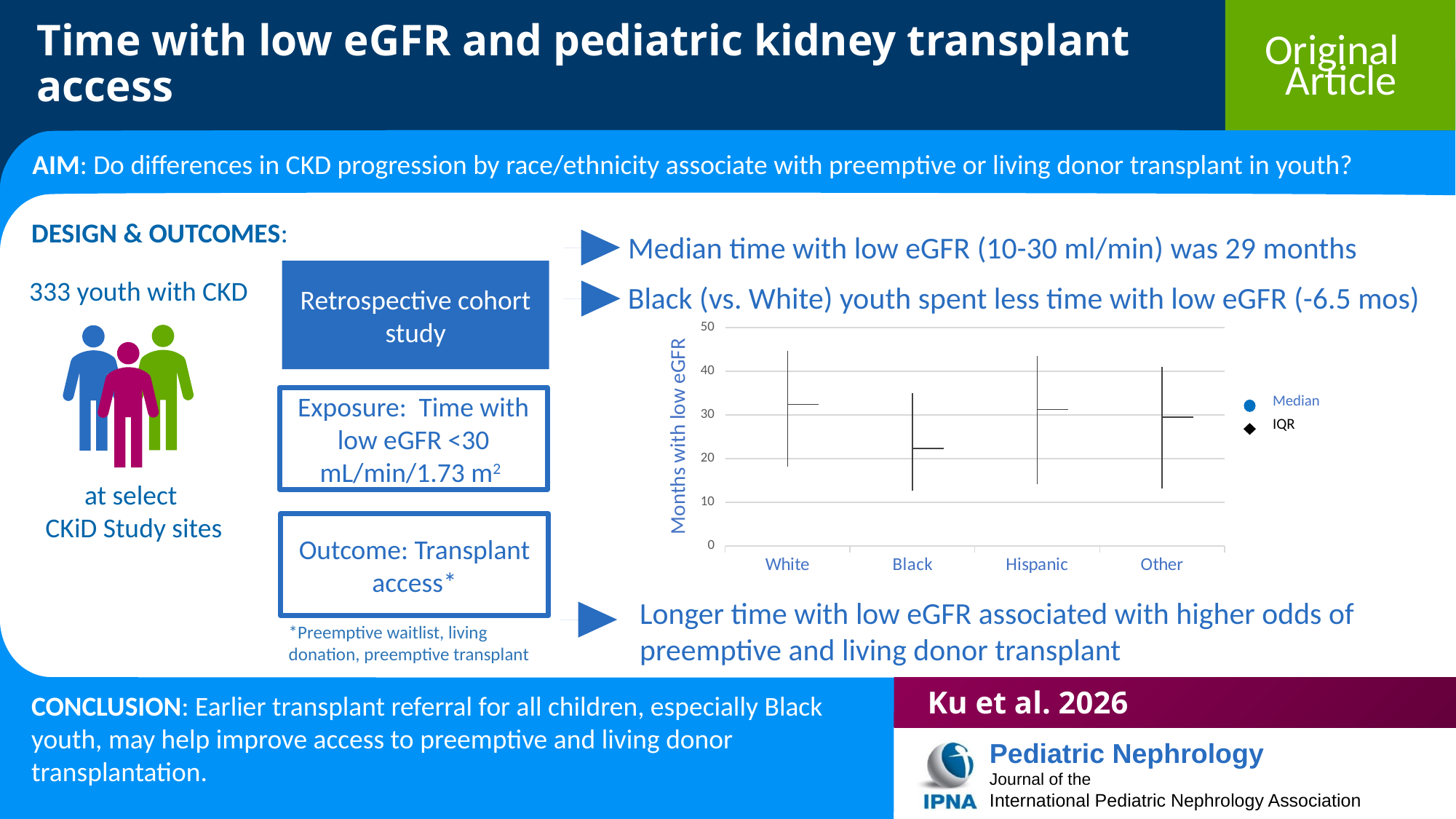

Time with low eGFR and pediatric kidney transplant access
AIM: Do differences in CKD progression by race/ethnicity associate with preemptive or living donor transplant in youth?
DESIGN & OUTCOMES:
Median time with low eGFR (10-30 ml/min) was 29 months
Retrospective cohort study
333 youth with CKD
Black (vs. White) youth spent less time with low eGFR (-6.5 mos)
[unsupported chart]
Months with low eGFR
Median
IQR
Exposure: Time with low eGFR <30 mL/min/1.73 m2
at select
CKiD Study sites
Outcome: Transplant access*
Longer time with low eGFR associated with higher odds of preemptive and living donor transplant
*Preemptive waitlist, living donation, preemptive transplant
Ku et al. 2026
CONCLUSION: Earlier transplant referral for all children, especially Black youth, may help improve access to preemptive and living donor transplantation.
