# Supplementary material for: Association between estimated time with low glomerular filtration rate and access to transplant among youth with advanced chronic kidney disease
Source: Pediatr Nephrol. 2026 Apr 6;41(9):2979–88. doi: 10.1007/s00467-026-07247-0 (PMC13178790; doi:10.1007/s00467-026-07247-0)
Supplement: Supplementary file 2 — (DOCX 29.2 KB) [file 467_2026_7247_MOESM2_ESM.docx]

**Supplemental Table 1.** List of principal site investigators of the Chronic Kidney Disease in Children (CKiD) cohort study.

| **Study Investigator(s)** | **Institution** | **City** | **State/Province** |
| --- | --- | --- | --- |
| Sahar Fathallah-Shaykh, MD | University of Alabama at Birmingham (Children’s of Alabama) | Birmingham | AL |
| Anjali Nayak, MD;  Gina-Marie Barletta, MD | Phoenix Children’s Hospital | Phoenix | AZ |
| Tom Blydt-Hansen, MD, FRCPC; Janis Dionne, MD, FRCPC | British Columbia Children’s Hospital | Vancouver | British Columbia, Canada |
| Cynthia Wong, MD | Stanford University Medical Center | Palo Alto | CA |
| Ora Yadin, MD | University of California – Los Angeles (UCLA) | Los Angeles | CA |
| Elizabeth Ingulli, MD; Robert Mak, MD, PhD | University of California – San Diego (UCSD) | San Diego | CA |
| Cheryl Sanchez-Kazi, MD | Loma Linda University | Loma Linda | CA |
| Asha Moudgil, MD | Children’s National Medical Center | Washington | DC |
| Sonal Bhatnagar, MD | Nemours/Alfred l. duPont Hospital for Children | Wilmington | DE |
| Carolyn Abitbol, MD; Marissa DeFrietas, MD; Chryso Katsoufis, MD; Wacharee Seeherunvong, MD | University of Miami | Miami | FL |
| Larry Greenbaum, MD, PhD | Children’s Healthcare of Atlanta / Emory University | Atlanta | GA |
| Lyndsay Harshman, MD | University of Iowa | Iowa City | IA |
| Priya Verghese, MBBS (MD), MPH | Ann & Robert H. Lurie Children’s Hospital of Chicago | Chicago | IL |
| Sonia Krishnan, MD | University of Illinois at Chicago | Chicago | IL |
| Amy Wilson, MD | Riley Hospital for Children at Indiana University Health | Indianapolis | IN |
| Stefan Kiessling, MD; Margaret Murphy, PhD | University of Kentucky | Lexington | KY |
| Siddharth Shah, MD; Janice Sullivan, MD | University of Louisville (Novak Center for Children’s Health) | Louisville | KY |
| Samir El-Dahr, MD; Stacy Drury, MD | Tulane University | New Orleans | LA |
| Nancy Rodig, MD | Boston Children’s Hospital | Boston | MA |
| Allison Dart, MD MSc, FRCPC | University of Manitoba (Children’s Hospital Research Institute of Manitoba) | Winnipeg | Manitoba, Canada |
| Meredith Atkinson, MD | Johns Hopkins University (Johns Hopkins Children’s Center) | Baltimore | MD |
| Zubin Modi, MD | University of Michigan | Ann Arbor | MI |
| Jason Thomas, MD | Spectrum Health Hospitals / Helen DeVos Children's Hospital | Grand Rapids | MI |
| Bradley Warady, MD | Children’s Mercy Hospital - Kansas City | Kansas City | MO |
| Rebecca Johnson, PhD | Children's Mercy Hospital | Kansas City | MO |
| Vikas Dharnidharka, MD | Washington University in St. Louis (St. Louis Children’s Hospital) | St. Louis | MO |
| Stephen Hooper, PhD | University of North Carolina | Chapel Hill | NC |
| Susan Massengill, MD | Levine Children’s Hospital | Charlottesville | NC |
| Liliana Gomez-Mendez, MD | East Carolina University | Greenville | NC |
| Matthew Hand, DO | Dartmouth-Hitchcock Medical Center | Lebanon | NH |
| Joann Carlson, MD | Rutgers-Robert Wood Johnson Medical School | New Brunswick | NJ |
| Craig Wong, MD, MPH | University of New Mexico Health Sciences Center | Albuquerque | NM |
| Frederick Kaskel, MD, PhD; Shlomo Shinnar, MD, PhD | Albert Einstein College of Medicine/Montefiore Medical Center | Bronx | NY |
| Jeffrey Saland, MD | Icahn School of Medicine at Mount Sinai | New York | NY |
| Rebecca Levy, MD; Marc Lande, MD; George Schwartz, MD | University of Rochester Medical Center | Rochester | NY |
| Anil Mongia, MD | State University of New York, Downstate Medical Center | Brooklyn | NY |
| Donna Claes, MD; Mark Mitsnefes, MD | Cincinnati Children’s Hospital Medical Center | Cincinnati | OH |
| Katherine MacRae Dell, MD | Case Western Reserve University/Cleveland Clinic Children’s | Cleveland | OH |
| Hiren Patel, MD | Nationwide Children’s Hospital | Columbus | OH |
| Ikuyo Yamaguchi, MD, PhD | Oklahoma University Health Sciences Center | Oklahoma City | OK |
| Rulan Parekh, MD | Hospital for Sick Children (Sick Kids) | Toronto | Ontario, Canada |
| Amira Al-Uzri, MD, MCR; Kelsey Richardson, MD | Oregon Health and Science University | Portland | OR |
| Susan Furth, MD, PhD; Larry Copelovitch, MD; Erum Hartung, MD, MTR | Children’s Hospital of Philadelphia | Philadelphia | PA |
| Elaine Ku, MD, MAS | University of California – San Francisco (UCSF) | San Francisco | SF |
| Joshua Samuels, MD | University of Texas Health Science Center at Houston | Houston | TX |
| Poyyapakkam Srivaths, MD | Baylor College of Medicine (Texas Children’s Hospital) | Houston | TX |
| Amy Becker, MD | Driscoll Children’s Hospital | Corpus Christi | TX |
| Davoud Mohtat, MD | INOVA Children’s Hospital / Pediatric Specialists of Virginia | Fairfax | VA |
| Victoria Norwood, MD | University of Virginia | Charlottesville | VA |
| Joseph Flynn, MD,  Susan Halbach, MD | Seattle Children’s Hospital | Seattle | WA |
| Rajasree Sreedharan, MD | Medical College of Wisconsin | Milwaukee | WI |
| Sharon Bartosh, MD | University of Wisconsin | Madison | WI |
