# Supplementary figures and images for: Association between estimated time with low glomerular filtration rate and access to transplant among youth with advanced chronic kidney disease
Source: Pediatr Nephrol. 2026 Apr 6;41(9):2979–88. doi: 10.1007/s00467-026-07247-0 (PMC13178790; doi:10.1007/s00467-026-07247-0)

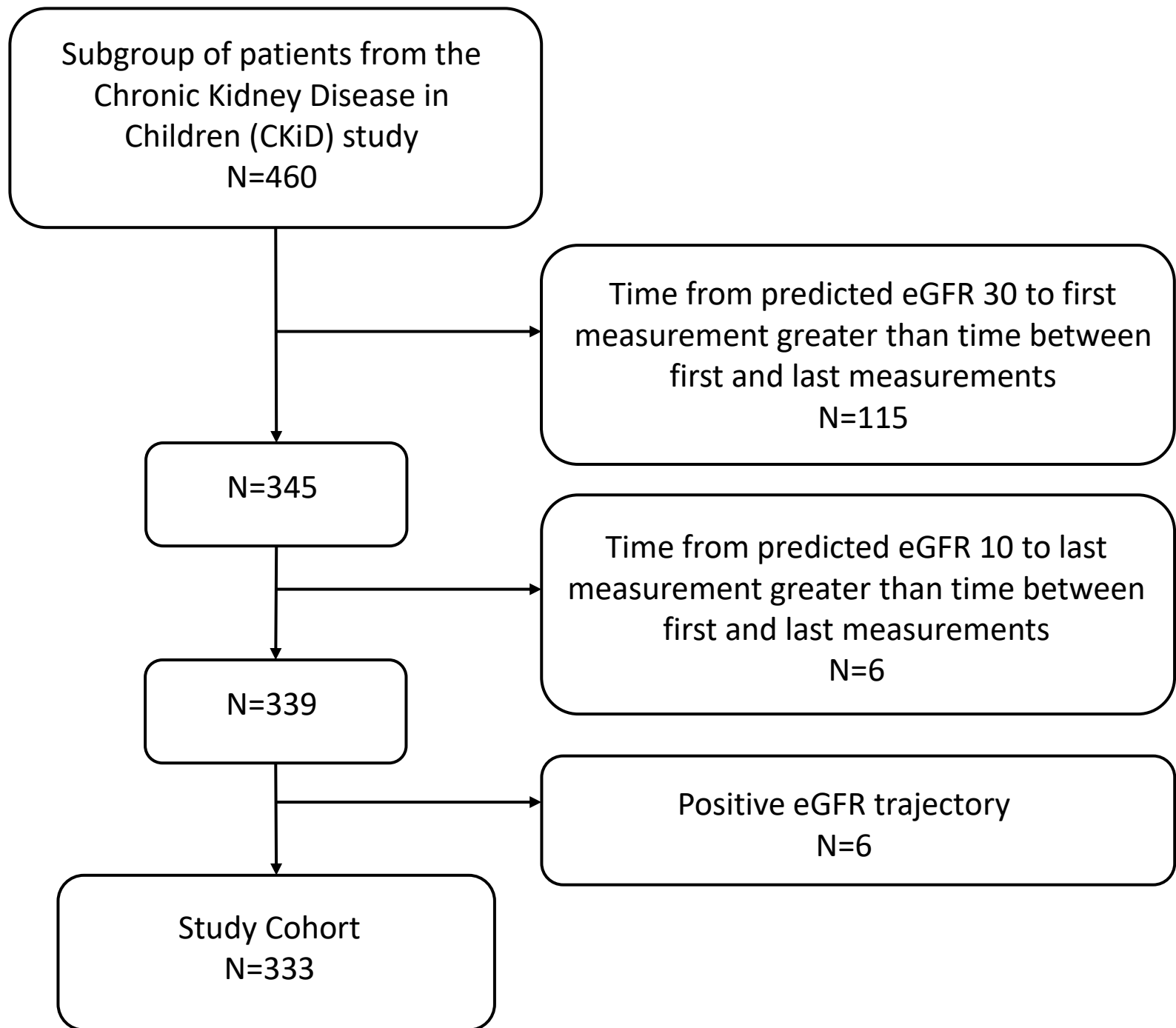

Supplement: Supplementary file 3 — Supplemental Fig. 1 Consort diagram (PDF 61.1 KB) [file 467_2026_7247_MOESM3_ESM.pdf]
